# Missed Opportunities for Prevention of Congenital Syphilis — United States, 2018

**DOI:** 10.15585/mmwr.mm6922a1

**Published:** 2020-06-05

**Authors:** Anne Kimball, Elizabeth Torrone, Kathryn Miele, Laura Bachmann, Phoebe Thorpe, Hillard Weinstock, Virginia Bowen

**Affiliations:** ^1^Epidemic Intelligence Service, CDC; ^2^Division of STD Prevention, National Center for HIV, Hepatitis, STD, and TB Prevention, CDC; ^3^Gilstrap Fellowship, CDC Foundation, Atlanta, Georgia.

Congenital syphilis is an infection with *Treponema pallidum* in an infant or fetus, acquired during pregnancy from a mother with untreated or inadequately treated syphilis. Congenital syphilis can cause miscarriage, stillbirth, or early infant death, and infected infants can experience lifelong physical and neurologic problems. Although timely identification and treatment of maternal syphilis during pregnancy can prevent congenital syphilis (*1*,*2*), the number of reported congenital syphilis cases in the United States increased 261% during 2013–2018, from 362 to 1,306. Among reported congenital syphilis cases during 2018, a total of 94 resulted in stillbirths or early infant deaths (*3*). Using 2018 national congenital syphilis surveillance data and a previously developed framework (*4*), CDC identified missed opportunities for congenital syphilis prevention. Nationally, the most commonly missed prevention opportunities were a lack of adequate maternal treatment despite the timely diagnosis of syphilis (30.7%) and a lack of timely prenatal care (28.2%), with variation by geographic region. Congenital syphilis prevention involves syphilis prevention for women and their partners and timely identification and treatment of pregnant women with syphilis. Preventing continued increases in congenital syphilis requires reducing barriers to family planning and prenatal care, ensuring syphilis screening at the first prenatal visit with rescreening at 28 weeks’ gestation and at delivery, as indicated, and adequately treating pregnant women with syphilis (*2*). Congenital syphilis prevention strategies that implement tailored public health and health care interventions to address missed opportunities can have substantial public health impact.

Congenital syphilis is a reportable condition in all 50 states and the District of Columbia and is nationally notifiable; case reports are sent voluntarily to CDC through the National Notifiable Diseases Surveillance System. According to the congenital syphilis surveillance case definition, congenital syphilis is 1) a condition affecting stillbirths and infants born to mothers with untreated or inadequately treated syphilis regardless of signs in the infant or 2) a condition affecting an infant with clinical evidence of congenital syphilis including direct detection of *Treponema pallidum* or a reactive nontreponemal syphilis test with signs on physical examination, radiographs, or cerebrospinal fluid analysis (*3*). Rates of congenital syphilis mirror rates of primary and secondary syphilis among women of reproductive age, which approximately doubled during 2014–2018 (*3*). Adequate maternal treatment is defined as completion of a penicillin-based regimen recommended for the mother’s stage of syphilis initiated ≥30 days before delivery (*2*). For this analysis, all congenital syphilis prevention opportunities are considered timely if they occurred ≥30 days before delivery, per the surveillance case definition (*3*).

Demographic and clinical characteristics of infants and their mothers were analyzed using Stata statistical software (version 11; StataCorp). On the basis of CDC’s congenital syphilis prevention framework, each congenital syphilis case was assigned to one of four mutually exclusive missed opportunity categories based on the mother’s prenatal care, testing, and treatment history: 1) lack of timely prenatal care with no timely syphilis testing; 2) lack of timely syphilis testing despite timely prenatal care; 3) lack of adequate maternal treatment despite a timely syphilis diagnosis;* or 4) late identification of seroconversion during pregnancy (identified <30 days before delivery). Cases that did not fall into one of the four main missed opportunity categories were categorized as either 1) having signs or symptoms of congenital syphilis despite maternal treatment completion or 2) unable to be classified because of insufficient information reported to CDC. Missed opportunities were quantified nationally by U.S. Census Bureau region^†^ and by race/ethnicity for the highest morbidity regions to identify the most important strategies to prevent congenital syphilis.

## Characteristics of Infants with Congenital Syphilis and Their Mothers

Among 1,306 congenital syphilis cases reported during 2018, 685 (52.5%) occurred in the South, 465 (35.6%) in the West, 103 (7.9%) in the Midwest, and 53 (4.1%) in the Northeast Census regions (Table 1). Nationally, 510 (39.1%) mothers of infants with congenital syphilis were non-Hispanic black (black); 411 (31.5%) were Hispanic; 286 (21.9%) were non-Hispanic white (white); and 99 (7.6%) were of another race/ethnicity (non-Hispanic American Indian/Alaska Native [29], non-Hispanic Asian/Pacific Islander [26], or non-Hispanic other or unknown [44]) (Table 1). Approximately half of mothers of infants with congenital syphilis in the Midwest (54.4%) and Northeast (56.6%) had early stages of syphilis (primary, secondary, or early non-primary non-secondary^§^), compared with those in the South (36.6%) and the West (36.8%). The percentage of congenital syphilis cases that were live-born and symptomatic (33.2% nationally) or stillborn (6.0% nationally) also varied by region.

**TABLE 1:** Demographic and clinical characteristics of infants with congenital syphilis and their mothers, by U.S. Census region* — United States, 2018

Characteristic	Census region No. (%^†^)				
	Total	South	West	Midwest	Northeast
**Race/Ethnicity of mother^§^**					
White	**286 (21.9)**	117 (17.1)	130 (28.0)	29 (28.2)	10 (18.9)
Black	**510 (39.1)**	346 (50.5)	86 (18.5)	54 (52.4)	24 (45.3)
Hispanic	**411 (31.5)**	200 (29.2)	194 (41.7)	6 (5.8)	11 (20.7)
American Indian/Alaska Native	**29 (2.2)**	2 (0.3)	23 (4.9)	4 (3.9)	0 (0)
Asian/Pacific Islander	**26 (2.0)**	3 (0.4)	17 (3.7)	5 (4.9)	1 (1.9)
Other/Unknown	**44 (3.4)**	17 (2.5)	15 (3.2)	5 (4.9)	7 (13.2)
**Maternal stage of syphilis**					
Primary or secondary	**108 (8.3)**	48 (7.0)	43 (9.2)	11 (10.7)	6 (11.3)
Early non-primary non-secondary	**400 (30.6)**	203 (29.6)	128 (27.5)	45 (43.7)	24 (45.3)
Unknown duration or late	**664 (50.8)**	317 (46.3)	283 (60.9)	43 (41.7)	21 (39.6)
Other/Missing	**134 (10.3)**	117 (17.1)	11 (2.4)	4 (3.9)	2 (3.8)
**Infant outcomes**					
Live-born with signs or symptoms of congenital syphilis^¶^	**434 (33.2)**	167 (24.4)	198 (42.6)	46 (44.7)	23 (43.4)
Live-born with no documented signs or symptoms of congenital syphilis	**788 (60.3)**	474 (69.2)	236 (50.8)	52 (50.5)	26 (49.1)
Stillborn	**78 (6.0)**	41 (6.0)	29 (6.2)	4 (3.9)	4 (7.5)
Unknown vital status	**6 (0.5)**	3 (0.4)	2 (0.4)	1 (1.0)	0 (0)
**Total**	**1,306**	**685**	**465**	**103**	**53**

* *South*: Alabama, Arkansas, Delaware, District of Columbia, Florida, Georgia, Kentucky, Louisiana, Maryland, Mississippi, North Carolina, Oklahoma, South Carolina, Tennessee, Texas, Virginia, and West Virginia; *West*: Alaska, Arizona, California, Colorado, Hawaii, Idaho, Montana, Nevada, New Mexico, Oregon, Utah, Washington, and Wyoming; *Midwest*: Illinois, Indiana, Iowa, Kansas, Michigan, Minnesota, Missouri, Nebraska, North Dakota, Ohio, South Dakota, and Wisconsin; *Northeast*: Connecticut, Maine, Massachusetts, New Hampshire, New Jersey, New York, Pennsylvania, Rhode Island, and Vermont.

^†^ Percentages might not sum to 100 because of rounding.

^§^ Whites, blacks, American Indians/Alaska Natives, Asians/Pacific Islanders, and others/unknown were non-Hispanic; Hispanics could be of any race.

^¶^ Signs or symptoms of congenital syphilis include any one of the following: condyloma lata, snuffles, syphilitic rash, hepatosplenomegaly, jaundice/hepatitis, pseudoparalysis, or edema on physical exam; long-bone radiograph findings consistent with congenital syphilis; abnormal protein or white blood cell count in the cerebrospinal fluid; reactive venereal disease research laboratory test in the cerebrospinal fluid; direct detection of *Treponema pallidum* by dark field microscopy or special stains.

## Missed Opportunities for Prevention

Nationally, the most commonly missed prevention opportunity was a lack of adequate maternal treatment despite the timely diagnosis of syphilis during pregnancy (30.7%), followed closely by a lack of timely prenatal care (28.2%) (Table 2). This national pattern was reflected in the South (lack of adequate treatment: 34.3%; lack of prenatal care: 19.9%). In the West, however, the most commonly missed opportunity was a lack of timely prenatal care (41.1%), followed by a lack of adequate maternal treatment despite a timely diagnosis (28.6%). In the Northeast, the most commonly missed opportunity was late identification of seroconversion during pregnancy (39.6%).

**TABLE 2:** Missed congenital syphilis prevention opportunities among mothers of infants with congenital syphilis, by U.S. Census region* — United States, 2018

Missed prevention opportunity	Census region No. (%^†^)				
	Total	South	West	Midwest	Northeast
No timely prenatal care and no timely syphilis testing	**368 (28.2)**	136 (19.9)	191 (41.1)	25 (24.3)	16 (30.2)
No timely syphilis testing despite receipt of timely prenatal care	**116 (8.9)**	47 (6.9)	55 (11.8)	8 (7.8)	6 (11.3)
No adequate maternal treatment despite a timely syphilis diagnosis	**401 (30.7)**	235 (34.3)	133 (28.6)	26 (25.2)	7 (13.2)
Late identification of seroconversion during pregnancy^§^	**146 (11.2)**	73 (10.7)	30 (6.5)	22 (21.4)	21 (39.6)
**Missed prevention opportunity not identified**					
Clinical evidence of congenital syphilis despite maternal treatment completion^¶^	**46 (3.5)**	33 (4.8)	9 (1.9)	4 (3.9)	0 (0.0)
Insufficient information**	**229 (17.5)**	161 (23.5)	47 (10.1)	18 (17.5)	3 (5.7)
**Total**	**1,306**	**685**	**465**	**103**	**53**

* *South*: Alabama, Arkansas, Delaware, District of Columbia, Florida, Georgia, Kentucky, Louisiana, Maryland, Mississippi, North Carolina, Oklahoma, South Carolina, Tennessee, Texas, Virginia, and West Virginia; *West*: Alaska, Arizona, California, Colorado, Hawaii, Idaho, Montana, Nevada, New Mexico, Oregon, Utah, Washington, and Wyoming; *Midwest*: Illinois, Indiana, Iowa, Kansas, Michigan, Minnesota, Missouri, Nebraska, North Dakota, Ohio, South Dakota, and Wisconsin; *Northeast*: Connecticut, Maine, Massachusetts, New Hampshire, New Jersey, New York, Pennsylvania, Rhode Island, and Vermont.

^†^ Percentages might not sum to 100 because of rounding.

^§^ Must have had a negative syphilis test early in pregnancy and a positive syphilis test <30 days before delivery, at day of delivery, or ≤90 days after delivery to be classified as having a seroconversion during pregnancy.

^¶^ Infant indications of infection include direct detection of *Treponema pallidum* by dark field microscopy or special stains; a reactive nontreponemal test and any one of these signs or symptoms of congenital syphilis: condyloma lata, snuffles, syphilitic rash, hepatosplenomegaly, jaundice/hepatitis, pseudoparalysis, or edema on physical exam; long-bone radiograph findings consistent with congenital syphilis; abnormal protein or white blood cell count in the cerebrospinal fluid; or reactive venereal disease research laboratory test in the cerebrospinal fluid.

** Insufficient information submitted to CDC related to maternal prenatal care, testing, or treatment to categorize.

Racial/ethnic disparities existed within the highest morbidity regions. In the South, the most commonly missed prevention opportunity among white mothers of infants with congenital syphilis was lack of timely prenatal care (31.6%), whereas among black and Hispanic mothers, lack of adequate maternal treatment (37.0%) was the most common (Table 3). In the West, racial/ethnic differences were less pronounced: regardless of race/ethnicity, >41% of mothers of infants with congenital syphilis lacked timely prenatal care, and >29% lacked adequate treatment despite receipt of a timely syphilis diagnosis.

**TABLE 3:** Missed congenital syphilis prevention opportunities among mothers of infants with congenital syphilis in the South and West U.S. Census regions,* by race/ethnicity^†^ — United States, 2018

Missed prevention opportunity	Census region and race/ethnicity No. (%^§^)					
	South			West		
	White	Black	Hispanic	White	Black	Hispanic
**No timely prenatal care and no timely syphilis testing**	**37 (31.6)**	**68 (19.7)**	**26 (13.0)**	**56 (43.1)**	**37 (43.0)**	**81 (41.8)**
No timely syphilis testing despite receipt of timely prenatal care	7 (6.0)	26 (7.5)	14 (7.0)	17 (13.1)	6 (7.0)	23 (11.9)
No adequate maternal treatment despite a timely syphilis diagnosis	28 (23.9)	128 (37.0)	74 (37.0)	38 (29.2)	26 (30.2)	57 (29.4)
Late identification of seroconversion during pregnancy^¶^	18 (15.4)	34 (9.8)	19 (9.5)	7 (5.4)	4 (4.7)	14 (7.2)
**Missed prevention opportunity not identified**						
Clinical evidence of congenital syphilis despite adequate maternal treatment completion**	5 (4.3)	17 (4.9)	9 (4.5)	3 (2.3)	2 (2.3)	2 (1.0)
Insufficient information^††^	22 (18.8)	73 (21.1)	58 (29.0)	9 (6.9)	11 (12.8)	17 (8.8)
**Total**	**117**	**346**	**200**	**130**	**86**	**194**

* *South*: Alabama, Arkansas, Delaware, District of Columbia, Florida, Georgia, Kentucky, Louisiana, Maryland, Mississippi, North Carolina, Oklahoma, South Carolina, Tennessee, Texas, Virginia, and West Virginia; *West*: Alaska, Arizona, California, Colorado, Hawaii, Idaho, Montana, Nevada, New Mexico, Oregon, Utah, Washington, and Wyoming.

^†^ White and black mothers were non-Hispanic; Hispanic mothers might be of any race.

^§^ Percentages might not sum to 100 because of rounding.

^¶^ Must have had negative syphilis test early in pregnancy and a positive syphilis test <30 days before delivery, at day of delivery, or ≤90 days after delivery to be classified as having a seroconversion during pregnancy.

** Infant indications of infection include direct detection of *Treponema pallidum* by dark field microscopy or special stains; a reactive nontreponemal test and any one of these signs or symptoms of congenital syphilis: condyloma lata, snuffles, syphilitic rash, hepatosplenomegaly, jaundice/hepatitis, pseudoparalysis, or edema on physical exam; long bone radiograph findings consistent with congenital syphilis; abnormal protein or white blood cell count in the cerebrospinal fluid; reactive venereal disease research laboratory test in the cerebrospinal fluid.

^††^ Insufficient information submitted to CDC related to maternal prenatal care, testing, or treatment to categorize.

## Discussion

Nationally, the most commonly missed opportunity for preventing congenital syphilis was lack of adequate maternal treatment, likely driven by the high numbers of cases in the South, where this missed opportunity was most prevalent. The most common missed opportunities for preventing congenital syphilis differed by geographic region. In the West, a lack of timely prenatal care was the most commonly missed opportunity, and in the Northeast, late identification of seroconversion was the most common. Regional clinical and demographic differences in mothers of infants with congenital syphilis indicate that different populations are at increased risk and might require different interventions. The high proportion of mothers with early syphilis in certain regions signals recent heterosexual transmission and the potential for future increases in congenital syphilis cases if no intervention occurs. The high proportions of symptomatic and stillborn infants in certain regions might be related to early syphilis among their mothers, given that higher rates of vertical transmission and worse infant outcomes are associated with early syphilis during pregnancy (*5*).

Published analyses of state-level data demonstrate additional heterogeneity in prevalences of missed opportunities and priority interventions. Repeat syphilis testing early in the third trimester was recently identified as the main intervention for preventing congenital syphilis in Florida, Louisiana, and New York City (*6*,*7*). A review of recent congenital syphilis cases in Indiana found that social vulnerabilities, including homelessness, substance abuse, and incarceration, were barriers to receiving timely diagnosis and treatment, despite provider adherence to CDC guidelines (*8*). A California study of missed opportunities for prevention of congenital syphilis identified gaps in multiple steps of the prevention cascade and found that early prenatal care is critical to preventing congenital syphilis and that multifaceted efforts are needed (*9*). Establishment of congenital syphilis case review boards in Louisiana identified specific missed opportunities, including lack of screening and treatment delay (*10*). These data support the need for tailored interventions based on local epidemiology and analysis of missed prevention opportunities.

A national congenital syphilis prevention strategy requires prioritizing interventions to address the root causes of missed opportunities while maximizing the impact of finite resources. Interventions are needed for identifying pregnant women with syphilis outside of prenatal care and for reducing barriers to prenatal care for all women. Ensuring timely follow-up of positive syphilis test results for pregnant women and reducing barriers to adequate syphilis treatment for pregnant women and their partners can prevent congenital syphilis cases. Syphilis screening for all pregnant women at the first prenatal visit with repeat screening at 28 weeks and at delivery for women in high prevalence areas or who are at increased risk for acquisition can further reduce congenital syphilis and its associated morbidity. These interventions require collaboration among public health authorities, health care organizations and providers, and policymakers. Jurisdictions can establish congenital syphilis case review boards that can identify local prevention failures and explore solutions. The differences in missed opportunities noted among regions and among racial/ethnic groups within regions demonstrate that tailored prevention efforts are needed.

The findings in this report are subject to at least three limitations. First, U.S. jurisdictions have different processes for congenital syphilis case investigation and reporting, and congenital syphilis investigations can be time-consuming and complicated. Inaccurate or incomplete data can lead to misclassification of missed prevention opportunity categories and might have magnified observed regional differences. Second, case report data provide limited information regarding each infant with congenital syphilis and each mother of an infant with congenital syphilis; this can lead to underascertainment of such factors as seroconversion. Finally, national congenital syphilis case report data do not contain information regarding social determinants of health such as maternal substance use; thus, this analysis cannot address the multifactorial barriers to accessing prenatal care and receiving adequate treatment.

Congenital syphilis prevention requires syphilis prevention for women and their sex partners and timely identification and treatment of pregnant women with syphilis. Improving access to prenatal care and family planning for all women can improve rates of congenital syphilis as well as many other maternal and child health outcomes. Regional differences in the missed prevention opportunities indicate a need for different priorities for interventions that address root causes of congenital syphilis. Halting the continued increases and eventually eliminating congenital syphilis in the United States will require collaboration between public health and health care sectors, understanding missed prevention opportunities, and implementing tailored interventions accordingly.

**Summary**

**What is already known about this topic?**

Timely identification and treatment of maternal syphilis can prevent congenital syphilis; however, the number of congenital syphilis cases in the United States increased 261% during 2013–2018.

**What is added by this report?**

Nationally, the most commonly missed opportunities for prevention of congenital syphilis are a lack of adequate maternal treatment despite timely diagnoses of syphilis (31%) and a lack of timely prenatal care (28%), followed by late identification of seroconversions (11%); prevalences of these missed opportunities differ regionally and by race/ethnicity.

**What are the implications for public health practice?**

Halting continued increases in congenital syphilis requires understanding the missed prevention opportunities and implementing tailored interventions based on local experience.

## Footnotes

* For a case of congenital syphilis to be categorized as resulting from this missed opportunity, a pregnant person would 1) need to have evidence of a diagnosis of syphilis during pregnancy with syphilis testing performed ≥30 days before delivery and 2) not have received adequate treatment for syphilis. Those who did not receive adequate treatment had no treatment at all, only received 1 dose when 3 doses were indicated based on maternal staging, received the doses at improper intervals, received the first dose of treatment <30 days before delivery, or were treated with a nonpenicillin–based regimen.

† U.S Census Bureau regions are Northeast, Midwest, South, and West. https://www2.census.gov/geo/pdfs/maps-data/maps/reference/us_regdiv.pdf.

§ Primary and secondary syphilis are early stages of syphilis marked by specific clinical characteristics and laboratory evidence. Early non-primary non-secondary syphilis (formerly known as early latent syphilis) is a stage of infection with *T. pallidum* in which the infection occurred within the previous 12 months, but there are no signs or symptoms of primary or secondary syphilis when the infection is identified. https://wwwn.cdc.gov/nndss/conditions/syphilis-early-non-primary-non-secondary/case-definition/2018/.
